# Supplementary material for: The type 1 diabetes gene TYK2 regulates β-cell development and its responses to interferon-α
Source: Nat Commun. 2022 Oct 26;13:6363. doi: 10.1038/s41467-022-34069-z (PMC9606380; doi:10.1038/s41467-022-34069-z)
Supplement: Supplementary file 1 — Supplementary Information [file 41467_2022_34069_MOESM1_ESM.pdf]

# The type 1 diabetes gene *TYK2* regulates β-cell development and its responses to interferon-α

Vikash Chandra[1#*], Hazem Ibrahim[1#], Clémentine Halliez[2], Rashmi B Prasad[3,4], Federica Vecchio[2], Om Prakash Dwivedi[4], Jouni Kvist[1], Diego Balboa[1,5], Jonna Saarimäki-Vire[1], Hossam Montaser[1], Tom Barsby[1], Väinö Lithovius[1], Isabella Artner[6], Swetha Gopalakrishnan[7], Leif Groop[4], Roberto Mallone[2,8], Decio L. Eizirik[9], Timo Otonkoski[1,10*]

## Supplementary Figures

- **Supplementary Fig. 1:** Generation and characterization of *TYK2* knockout hiPSCs clones.
- **Supplementary Fig. 2:** *In vitro* characterization of WT and *TYK2* KO hiPSCs at early stages of pancreatic differentiation.
- **Supplementary Fig. 3:** *In vitro* characterization of WT and *TYK2* KO S7 SC-islets for α-like cells ($GCG^+$), δ-like cells ($SST^+$) and proliferating cells (Ki-$67^+$).
- **Supplementary Fig. 4:** *In vivo* functional assessment of implanted WT and *TYK2* KO S7 SC islets.
- **Supplementary Fig. 5:** TYK2 inhibitor (TYK2i; BMS-986165) treatment during early pancreatic differentiation compromised endocrine precursors formation in hESCs line H1.
- **Supplementary Fig. 6:** Quality assessment of scRNA-seq analysis and relative percentage of cells in noted clusters.
- **Supplementary Fig. 7:** SC-islets exposed to IFNα show similar global transcriptomic changes compared to human islet samples under similar treatment.
- **Supplementary Fig. 8:** TYK2 regulates IFNα stimulated genes and autoantigen (*GAD1* and *GAD2*) in SC-islets.
- **Supplementary Fig. 9:** TYK2 regulates MHC Class II expression in IFNα induced SC-islets.
- **Supplementary Fig. 10:** rs34536443 and rs2304256 SNPs protect against autoimmune diseases in the Finnish population.

## Supplementary Tables

- **Supplementary Table 1:** Cases and controls of *TYK2* rs34536443 and rs2304256 SNPs in R7 FinnGen dataset
- **Supplementary Table 2:** Guides and primers used for hiPSCs genome editing
- **Supplementary Table 3:** Differentiation protocol and media formulations
- **Supplementary Table 4:** Chemicals and Reagents used in the study
- **Supplementary Table 5:** Antibodies used for western blot
- **Supplementary Table 6:** Antibodies used for flow cytometry and immunofluorescence

## Supplementary Figures

Supplementary Fig. 1

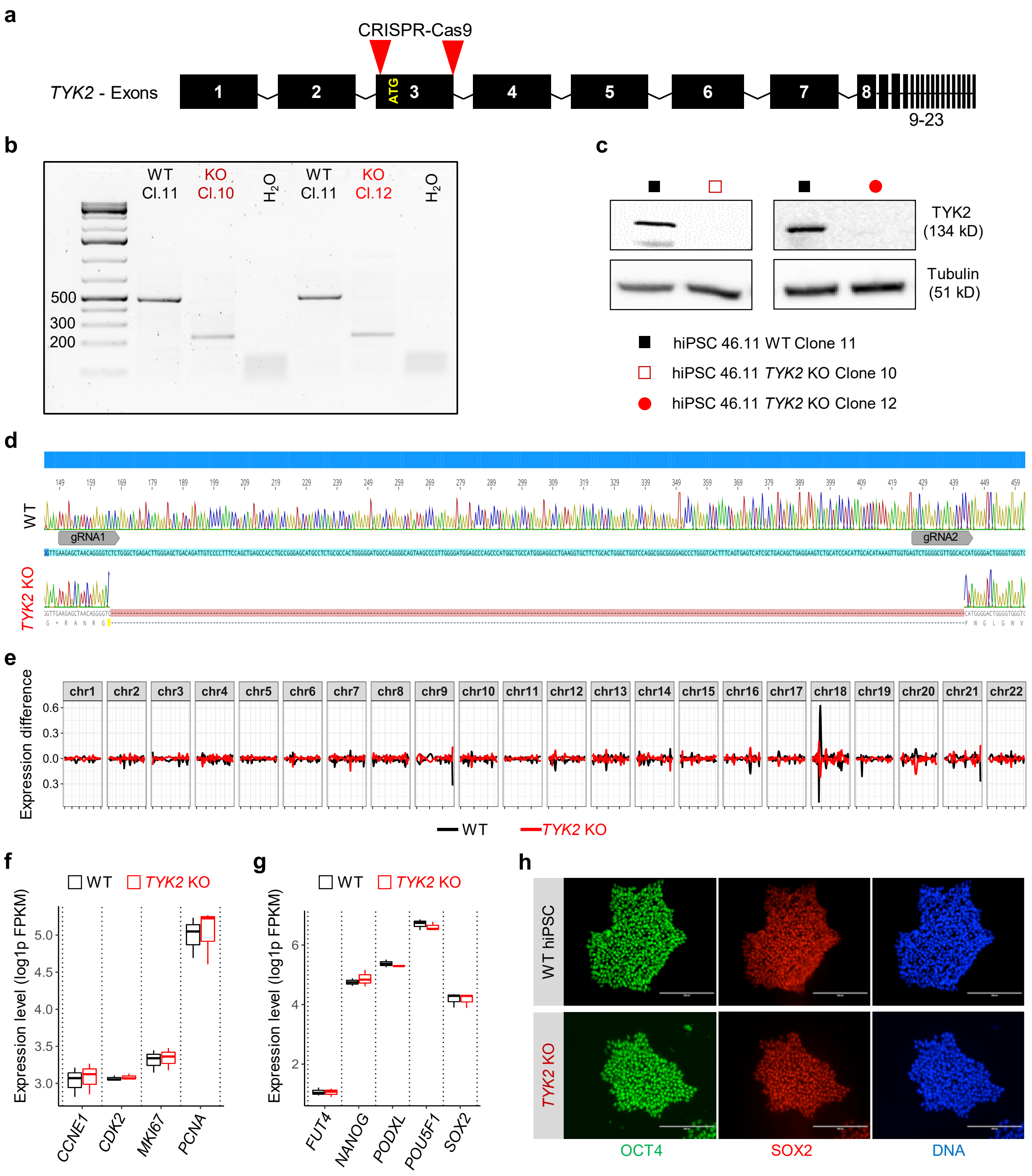

**Supplementary Fig. 1:** Generation and characterization of *TYK2* knockout hiPSCs clones. **(a)** Schematic for the strategy to generate *TYK2* knockout by removing the ATG-containing third exon in the *TYK2* gene of hiPSCs line HEL46.11. **(b)** A 504 bp PCR amplicon of *TYK2* gene showing a 277 bp deletion in KO clones (n = 3). **(c)** Immunoblot analysis for the expression of TYK2 protein in WT and *TYK2* KO hiPSC clones; tubulin protein expression shown for the loading control (n = 3). **(d)** Sanger sequencing confirming the exact 277 bp deletion in the KO C10 clone compared to the unedited WT clone C11 sequence. **(e)** Global gene expression-based e-karyotyping analysis for WT and *TYK2* KO hiPSCs lines (n = 12). **(f)** Boxplot showing the normalized FPKM values for the expression levels of proliferation markers, *CCNE1*, *CDK2*, *MKI67*, *PCNA* and **(g)** pluripotency factors, *FUT4*, *NANOG*, *PODXL*, *POU5FI*, *SOX2* utilizing whole transcriptome data of WT and *TYK2* KO hiPSCs cell-lines. The boxplots showing the median with lower and upper hinges corresponding to the first and third quartiles (the 25th and 75th percentiles), the whiskers extend from min to max values (n = 3). **(h)** Representative image of immunocytochemistry analysis for the expression of pluripotency factors OCT4 and SOX2 in WT and *TYK2* KO hiPSCs (n = 3). Scale bar = 200 μm. Source data are provided as a Source Data file.

Supplementary Fig. 2

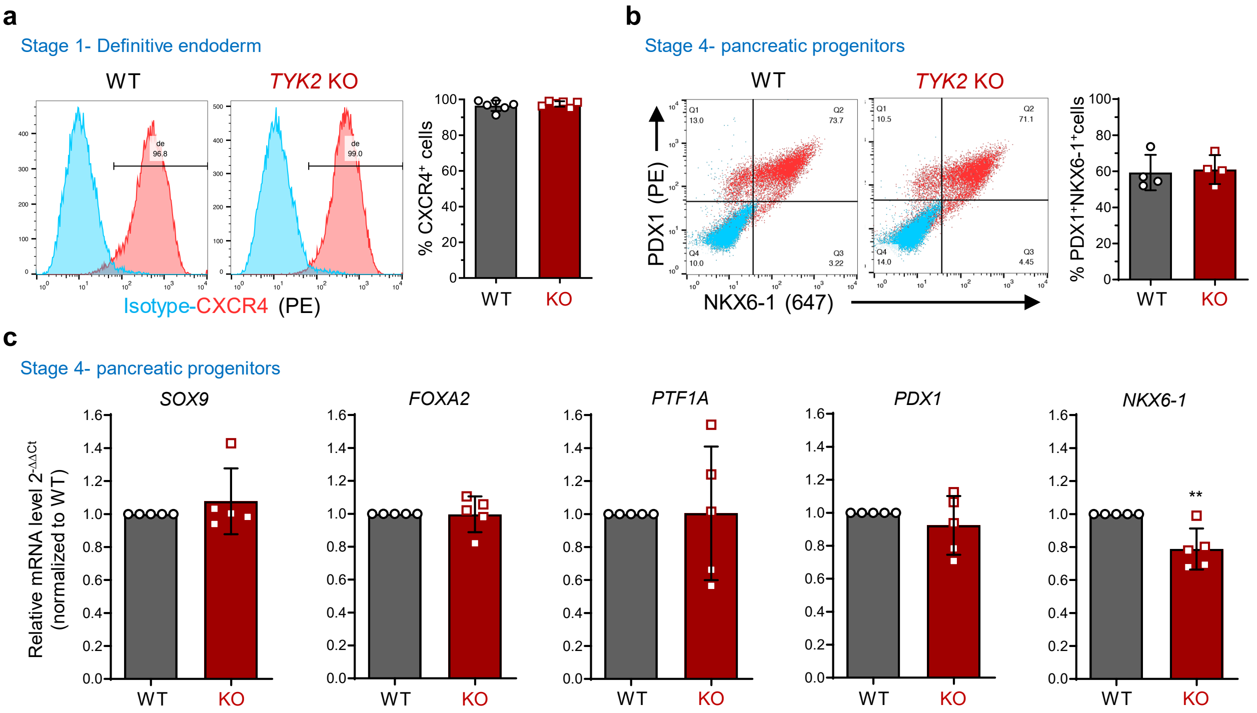

**Supplementary Fig. 2:** *In vitro* characterization of WT and *TYK2* KO hiPSCs at early stages of pancreatic differentiation.

**(a)** Flow cytometry analysis for the expression of $CXCR4^+$ definitive endoderm cells at the end of stage-1 and quantification for WT and *TYK2* KO cells (n = 6). **(b)** Flow cytometry analysis for $PDX1^+$ and $NKX6\text{-}1^+$ double positive cells at the end of stage-4 and quantification for WT and *TYK2* KO cells (n = 4). **(c)** Relative transcript expression of stage-4 pancreatic progenitor markers *SOX9*, *FOXA2*, *PTF1A*, *PDX1* and *NKX6-1* with qRT-PCR (n = 5). Two-tailed unpaired t-test was performed to determine the significance levels $^{**}p < 0.01$. Source data are provided as a Source Data file.

Supplementary Fig. 3

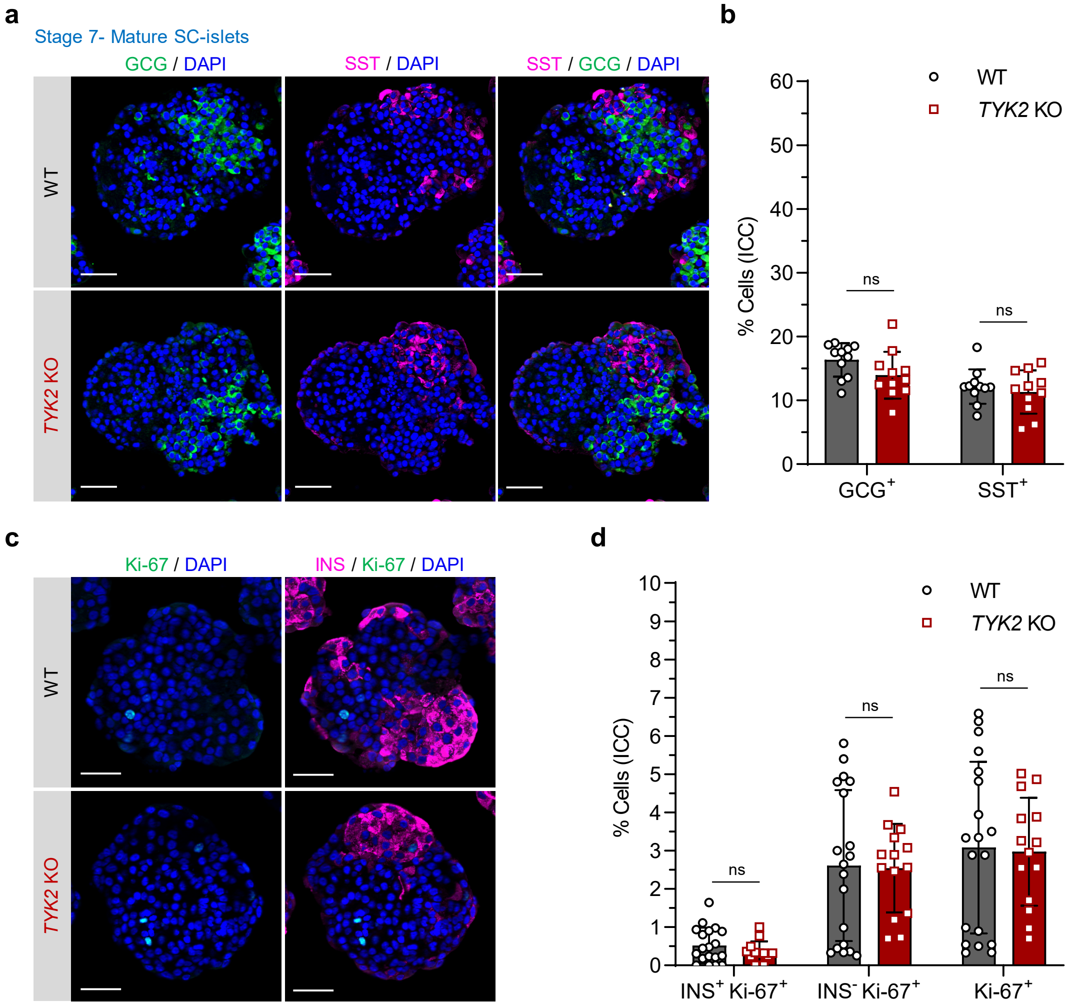

**Supplementary Fig. 3:** *In vitro* characterization of WT and *TYK2* KO S7 SC-islets for α-like cells ($GCG^+$), δ-like cells ($SST^+$) and proliferating cells (Ki-$67^+$).

**(a)** Representative image of immunocytochemistry for GCG and SST in S7 SC-islets (scale bar = 50 µm) and **(b)** their quantification from 2 experiments with 5-6 images each (n = 11). **(c)** Representative image of immunocytochemistry for Ki-67 and INS in S7 SC-islets (scale bar = 50 µm) and **(d)** their quantification from 2 experiments with 7-10 images each (n =14-19). Two-tailed unpaired t-test was performed to determine the significance levels, ns – non-significant. Data are presented as means ± S.D. Source data are provided as a Source Data file.

Supplementary Fig. 4

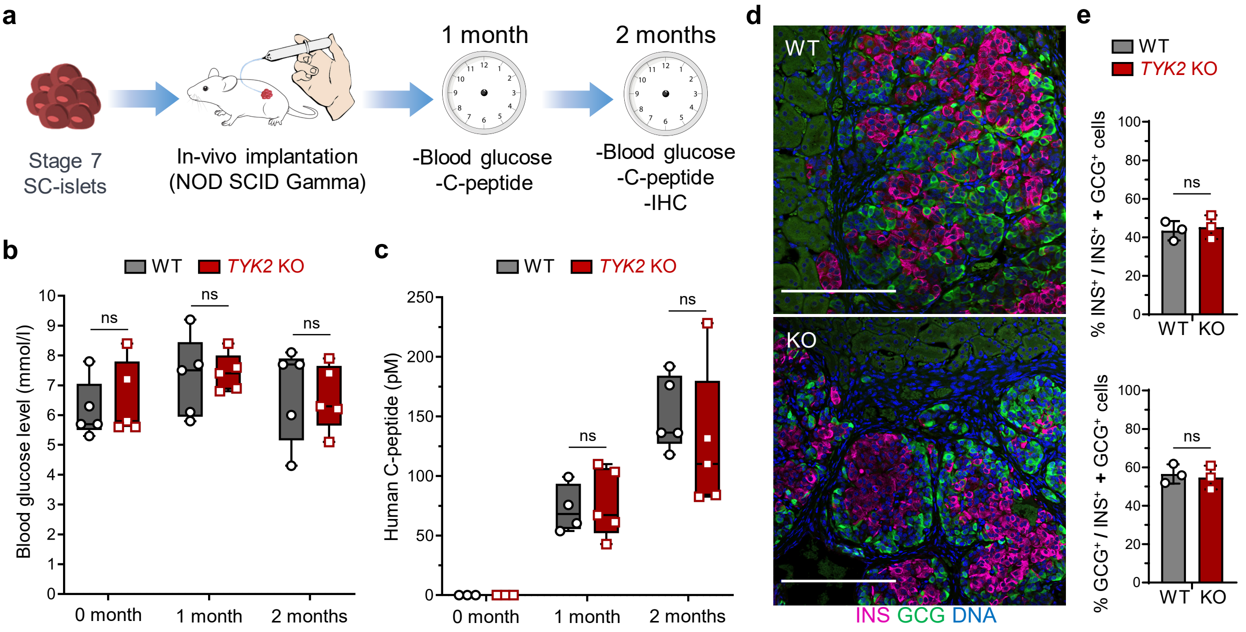

**Supplementary Fig. 4:** *In vivo* functional assessment of implanted WT and *TYK2* KO S7 SC-islets.

**(a)** Schematic of *in vivo* implantation study in NOD/SCID-γ mice. S7 WT and *TYK2* KO SC-islets were implanted in the mice (n = 5). The figure was partly generated using Servier Medical Art. **(b)** Mouse blood glucose levels measured at day 0, 1- and 2-months following implantation (n = 5). **(c)** Human C-peptide levels measured in the mice sera at day 0, 1- and 2-months following implantation (n = 3-5). Box and whiskers plots showing the median with min to max whiskers with all the points. **(d)** Immunohistochemistry of grafts after 2 months of implantation showing insulin (INS) and glucagon (GCG), scale bar = 200 μm and **(e)** their quantification for relative abundance ($INS^+$ or $GCG^+$ / $INS^+$ and $GCG^+$) in the graft sections (n = 3). Two-tailed unpaired t-test was performed to determine the significance levels. Data are mean ± S.D, ns – non-significant. Source data are provided as a Source Data file.

Supplementary Fig. 5

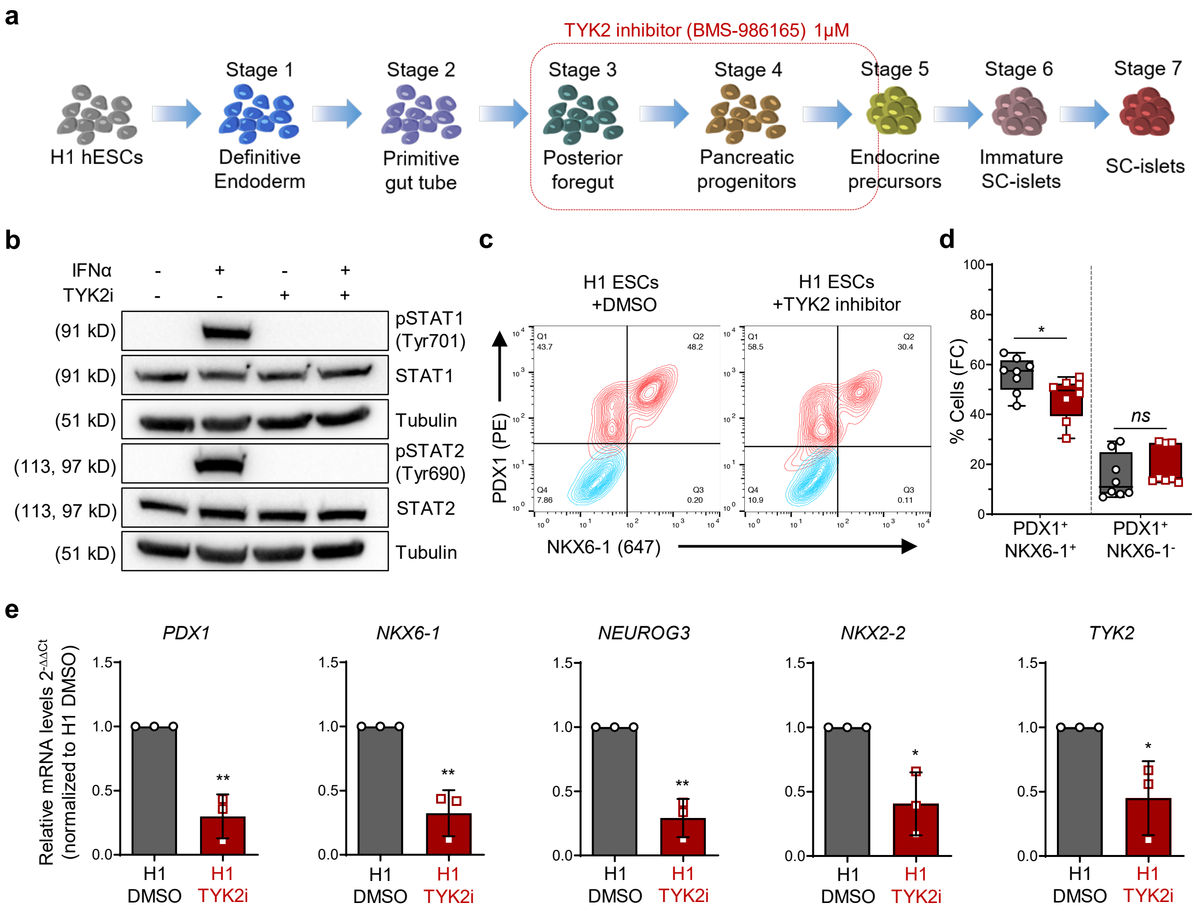

**Supplementary Fig. 5:** TYK2 inhibitor (TYK2i; BMS-986165) treatment during early pancreatic differentiation compromised endocrine precursors formation in hESCs H1 cell-line. **(a)** Human ES cell-line H1 differentiated with TYK2i as per schematic of the experiment plan. The figure was partly generated using Servier Medical Art. **(b)** Phosphorylation of STAT1 and STAT2 following 30 min IFNα treatments in DMSO or TYK2i treated differentiated cells (n = 3) at stage 5. **(c-d)** Representative contour plots of flow cytometry analysis following TYK2i treatment for the determination of $PDX1^{+}NKX6\text{-}1^{+}$ and $PDX1^{+}NKX6\text{-}1^{-}$ cells and their quantification (n = 8). Box and whiskers plot showing the median with min to max whiskers with all the points. **(e)** Relative transcript expression of *PDX1*, *NKX6-1*, *NEUROG3*, *NKX2-2* and *TYK2* with qRT-PCR (n = 3). Two-tailed unpaired t-test was performed to determine the significance levels. Data are mean ± S.D. *p < 0.05; **p < 0.01; ns – non-significant. Source data are provided as a Source Data file.

Supplementary Fig. 6

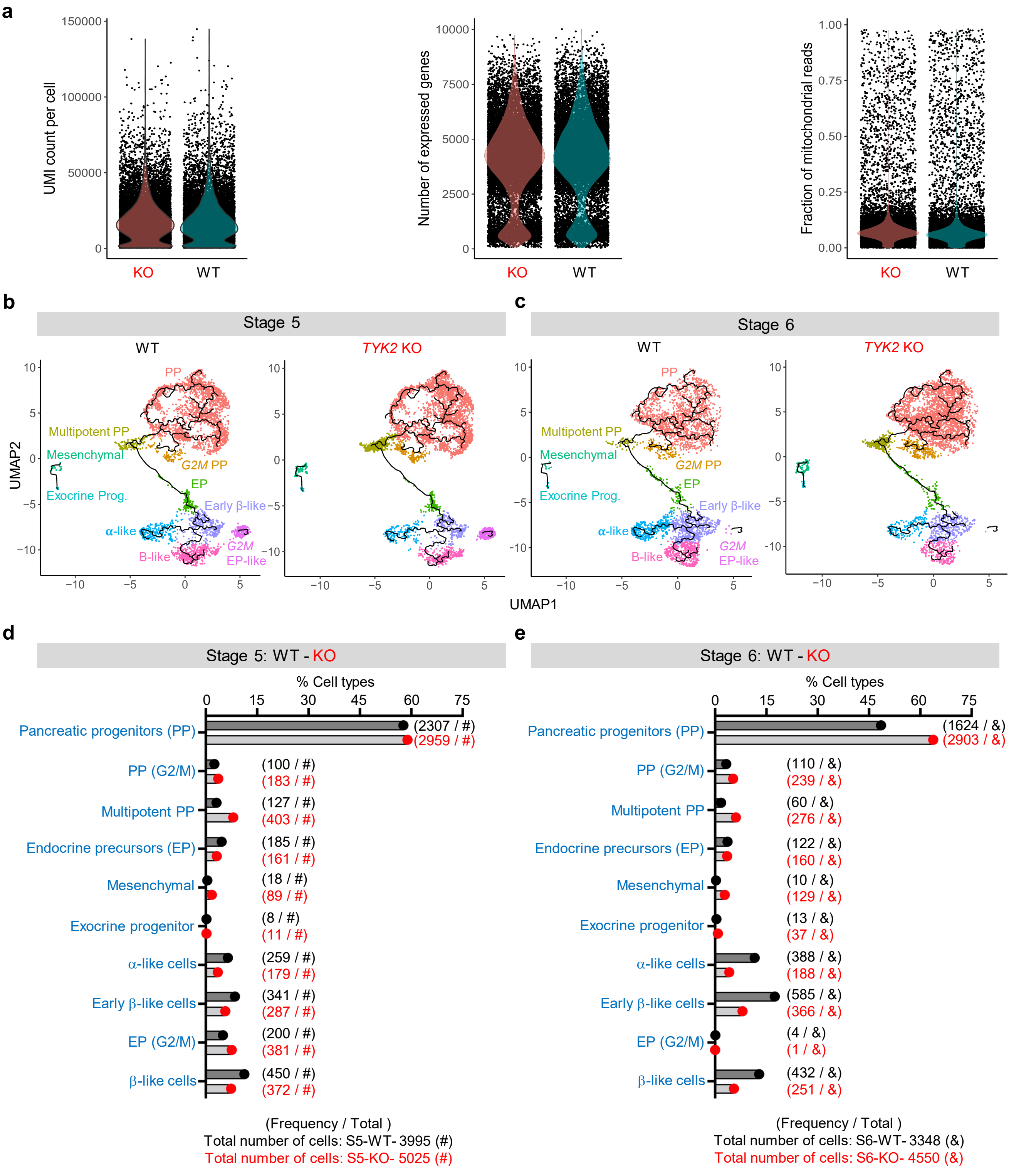

**Supplementary Fig. 6:** Quality assessment of scRNA-seq analysis and relative percentage of cells in noted clusters.

**(a)** Average unique molecular identifier (UMI) counts, number of expressed genes and mitochondrial reads per cell were plotted for the scRNA-seq analysis. Cells with less than 5000 UMI counts or 1700 expressed genes or with unusually high levels of mitochondrial reads (>20% of counts) were excluded. **(b)** Various noted clusters are indicated in different colour codes and presented as Uniform Manifold Approximation and projection (UMAP) with pseudotime trajectories for S5 and **(c)** S6 of WT and *TYK2* KO samples. **(d)** Relative percentage of cells in all noted clusters at S5 and **(e)** S6. Source data are provided as a Source Data file.

Supplementary Fig. 7

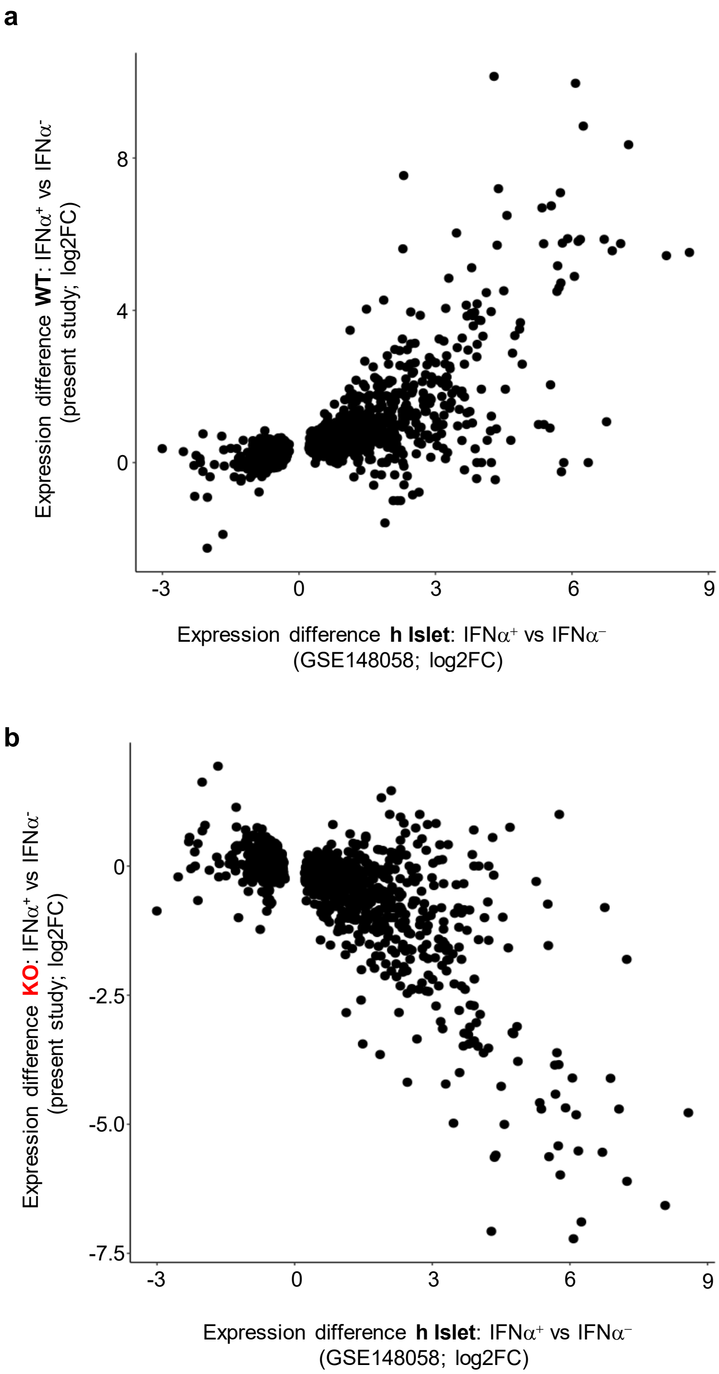

**Supplementary Fig. 7:** SC-islets exposed to IFNα show similar global transcriptomic changes compared to human islet samples under similar treatment.

Log2FC of the differentially expressed genes from a previous study (GSE148058) of human islets treated with or without IFNα (-/+) for 18h at X-axis versus the values of **(a)** WT or **(b)** *TYK2* KO SC-islets, treated for 24h with IFNα in the present study at Y-axis, respectively.

Supplementary Fig. 8

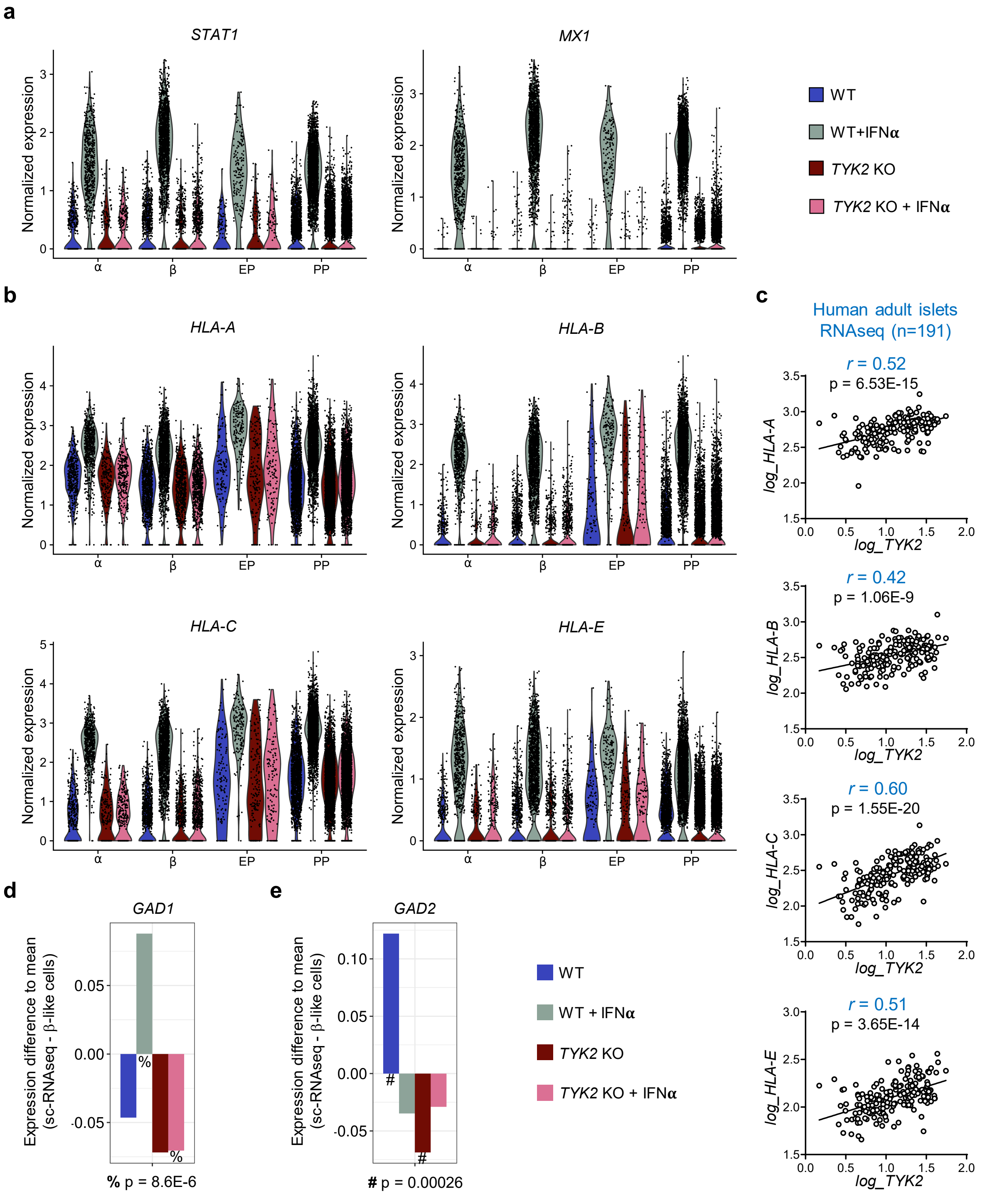

**Supplementary Fig. 8:** TYK2 regulates IFNα stimulated genes and autoantigen (*GAD1* and *GAD2*) in SC-islets.

Single cell transcriptomics performed on the WT and *TYK2* KO S6 SC-islets following 24h -/+ IFNα treatment. Violin plots showing the normalized expression of IFNα stimulated genes **(a)** *STAT1* and *MX1*, **(b)** *HLA-A*, *HLA-B*, *HLA-C* and *HLA-E* in the clusters of α-, β-, endocrine precursor (EP)- and pancreatic progenitor (PP)- like cells. **(c)** Gene expression correlation between *TYK2* and *HLA-A*, *HLA-B*, *HLA-C*, *HLA-E* with human islets RNA-seq samples (n = 191). Pearson's correlations *r* after log normalization of counts and significance levels *p* are indicated in the panel. **(d-e)** The Bar plots showing the expression pattern for the autoantigens *GAD1* and *GAD2* in different genotypes indicated by colour codes. Source data are provided as a Source Data file.

Supplementary Fig. 9

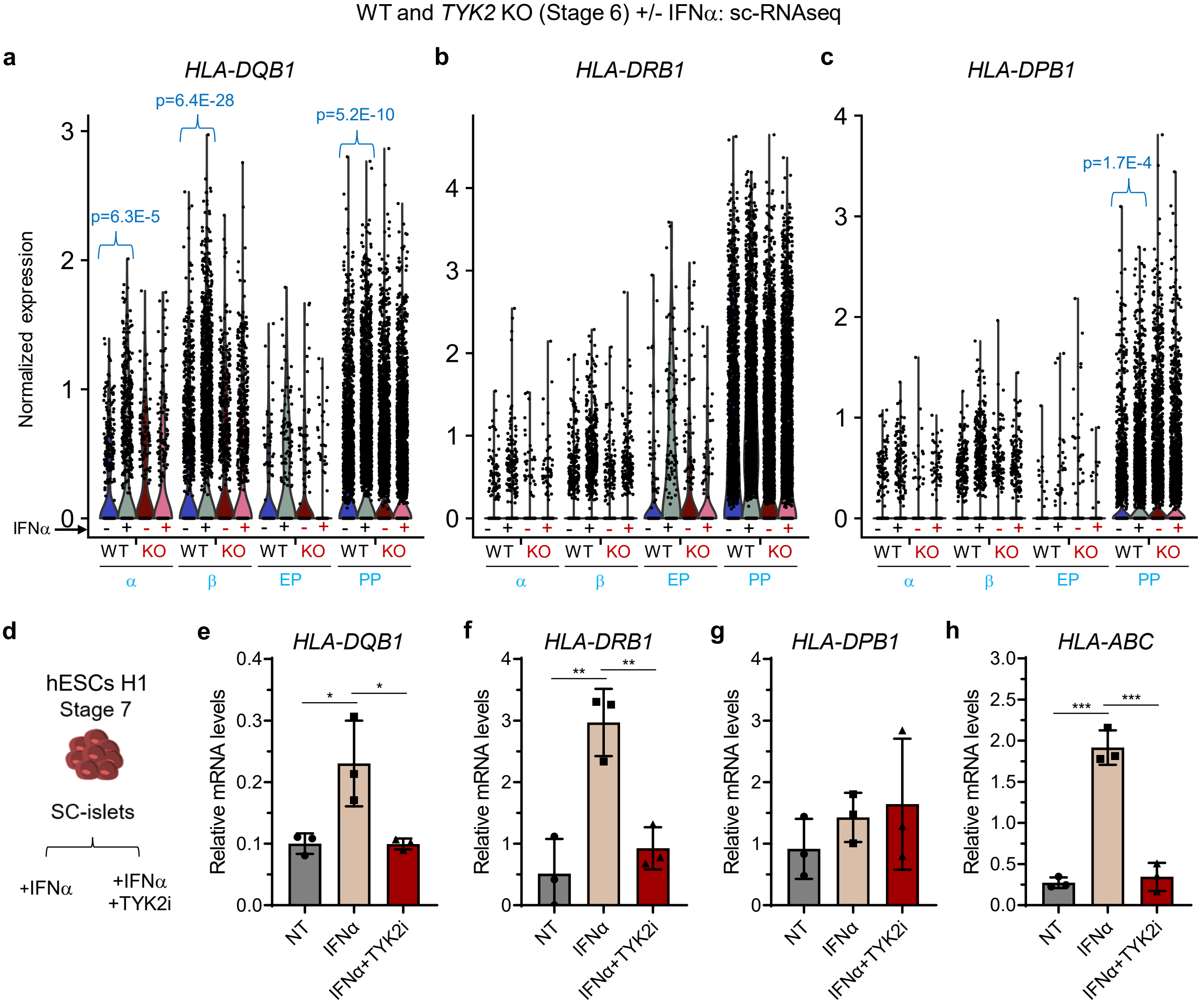

**Supplementary Fig. 9:** TYK2 regulates MHC Class II expression in IFNα induced SC-islets. **(a)** scRNA-seq based analysis for the expression of MHC Class II genes *HLA-DQB1* **(b)** *HLA-DRB1*, **(c)** *HLA-DPB1* upon IFNα treatment in S6 WT and *TYK2* KO cells. **(d)** Schematic of qRT-PCR based experiment to assess the expression of MHC Class II genes **(e)** *HLA-DQB1*, **(f)** *HLA-DRB1*, **(g)** *HLA-DPB1* and MHC Class I gene **(h)** *HLA-ABC* in matured S7 H1 SC-islets in presence of IFNα or IFNα+TYK2i (BMS-986165). Bar plots are means ± S.D. Ordinary one-way ANOVA with Tukey's multiple comparison (n = 3). *$p < 0.05$; **$p < 0.01$; ***$p < 0.001$. Source data are provided as a Source Data file.

Supplementary Fig. 10

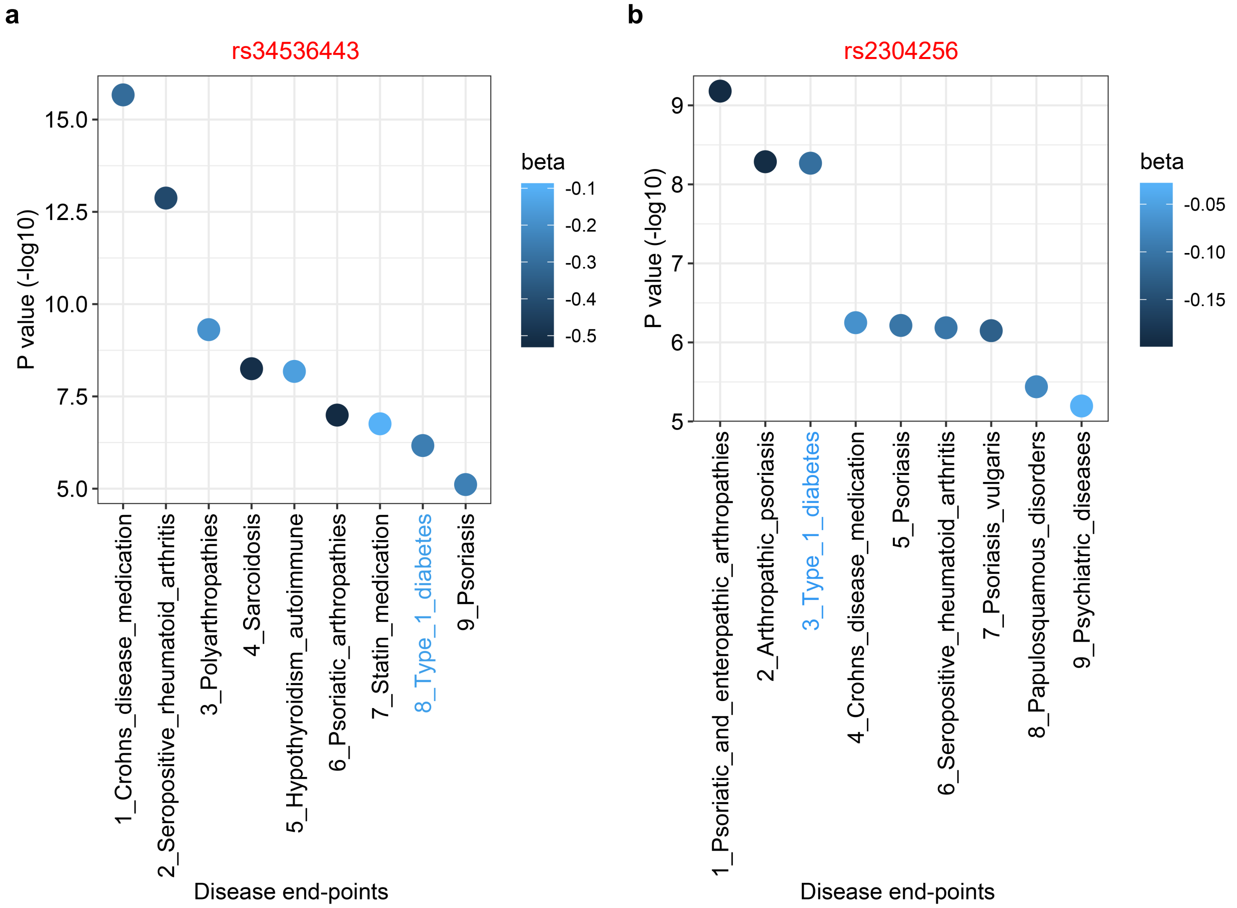

**Supplementary Fig. 10:** rs34536443 and rs2304256 SNPs protect against autoimmune diseases in the Finnish population.

The association analysis of **(a)** rs34536443 and **(b)** rs2304256 with 3095 clinical endpoints obtained from electronic health record data (ICD codes and drug purchase data) of 309,154 Finnish individuals. The figure depicts the effect size (beta) and the corresponding significance of association for the top ($p < 1.6x10^{-5}$) associated unique clinical endpoints (Supplementary Table 1). The association analysis was done using mixed model logistic regression adjusted for age, sex, 10 PCs and genotype batch. Source data are provided as a Source Data file.

## Supplementary Tables

**Supplementary Table 1:** Cases and controls of *TYK2* rs34536443 and rs2304256 SNPs in R7 FinnGen dataset

| | Phenotype | P_value (-log10) | beta | n_case | n_control | SNP |
|---|---|---|---|---|---|---|
| 1 | Crohn's disease medication | 15.6687003 | -0.303929 | 15801 | 293353 | rs34536443 |
| 2 | Seropositive rheumatoid arthritis | 12.8730994 | -0.415097 | 7294 | 238484 | |
| 3 | Polyarthropathies | 9.3086497 | -0.187344 | 24310 | 202617 | |
| 4 | Sarcoidosis | 8.25059997 | -0.505354 | 3103 | 304494 | |
| 5 | Hypothyroidism autoimmune | 8.17758999 | -0.150063 | 33422 | 227415 | |
| 6 | Psoriatic arthropathies | 6.99376079 | -0.520263 | 2430 | 202617 | |
| 7 | Statin medication | 6.75800054 | -0.0972771 | 100639 | 208515 | |
| 8 | Type 1 diabetes | 6.16889972 | -0.248286 | 8671 | 255466 | |
| 9 | Psoriasis | 5.11218023 | -0.241221 | 6995 | 299128 | |
| 1 | Psoriatic and enteropathic arthropathies | 9.17987016 | -0.195162 | 2818 | 238484 | rs2304256 |
| 2 | Arthropathic psoriasis | 8.28646998 | -0.192696 | 2571 | 299128 | |
| 3 | Type 1 diabetes | 8.26789989 | -0.107933 | 8671 | 255466 | |
| 4 | Crohn's disease medication | 6.24964977 | -0.067565 | 15801 | 293353 | |
| 5 | Psoriasis | 6.21471004 | -0.099716 | 6995 | 299128 | |
| 6 | Seropositive rheumatoid arthritis | 6.18526975 | -0.0991523 | 7294 | 238484 | |
| 7 | Psoriasis vulgaris | 6.14856002 | -0.125926 | 4321 | 299128 | |
| 8 | Papulosquamous disorders | 5.43886998 | -0.0775879 | 10026 | 299128 | |
| 9 | Psychiatric diseases | 5.19538018 | -0.0318792 | 79207 | 229947 | |

The association analysis was done using mixed model logistic regression adjusted for age, sex, 10 PCs and genotype batch.

**Supplementary Table 2:** Guides and primers used for hiPSCs genome editing

| Primers | Forward | Reverse |
|---|---|---|
| TYK2 PCR amp | GAGAGGAGGGTTCAGTGTTATTG | AGCTGCTGCTTGCACAC |
| TYK2 G1 OFF-TARGET1 | CTTGGGCTAGTAGGCGTGGC | GGGACACCCTGATCCCCAGT |
| TYK2 G1 OFF-TARGET2 | GAGGCAGGCAAGAAACCCCA | TGGGAGCCAGGAAGACTCACA |
| TYK2 G1 OFF-TARGET3 | TCACCGTGGAGCCAAAGTGT | AGGTGGGGCCAATCATCAGG |
| TYK2 G2 OFF-TARGET1 | GCAAAGTCAGAATCTGAGGGCA | TGTGAGAAAGCTGCTGGGTGA |
| TYK2 G2 OFF-TARGET2 | AGTTTGAAGTGGCTCTGGACCA | TGCACACCTTTATACTGGAAAAGAG |
| TYK2 G2 OFF-TARGET3 | GCCTTTGGCCAGCTCAAATG | TGGCCAATCCAGATGTCCCC |

**Supplementary Table 3:** Differentiation protocol and media formulations

| Stage | Reagent | Medium |
|---|---|---|
| Stage 1<br>3 days | d0: 100 ng/ml human Activin A, 3 μM CHIR<br>d1: 100 ng/ml human Activin A, 0.3 μM CHIR<br>d2: 100 ng/ml human Activin A | Basal 1: MCDB131 (10372-019, Life Technologies), 2 mM Glutamax, 1.5 g/L NaHCO3 (Sigma-Aldrich), 0.5% BSA fraction V Fatty acid free (Sigma-Aldrich), 10 mM glucose (Sigma-Aldrich) |
| Stage 2<br>3 days | 0.25 mM Ascorbic acid, 50 ng/ml FGF7 | |
| Stage 3<br>2 days | 0.25 mM Ascorbic acid, 50 ng/ml FGF7, 0.25 μM SANT1, 1 μM Retinoic Acid, 100 nM LDN, 200 nM and TPB | Basal 2: MCDB131, 2 mM Glutamax (35050038, Life Technologies), 2.5 g/L NaHCO3, 2% BSA fV, 10 mM glucose, 1:200 ITS-X (51500-056, Life Technologies) |
| Stage 4<br>4 days | 0.25 mM Ascorbic acid, 50 ng/ml FGF7, 0.25 μM SANT1, 0.1 μM Retinoic Acid, 200 nM LDN, 100 ng/ml EGF and 10 mM Nicotinamide | |
| Stage 5<br>4 days | 0.25 μM SANT1, 0.05 μM Retinoic acid, 100 nM LDN, 10 μM ALK5inhII, 1 μM GC1, 20 ng/mL Betacellulin and 100 nM GSiXX | Basal 3: MCDB131, 2 mM Glutamax, 1.5 g/L NaHCO3, 2% BSA fV, 20 mM glucose, 1:200 ITS-X, 10 μg/mL Heparin (H3149, Sigma-Aldrich), 10 μM Zinc Sulfate (Z0251, Sigma-Aldrich) |
| Stage 6<br>8 days | 100 nM LDN, 10 μM ALK5inhII, 1 μM GC1 and 100 nM GSiXX | |
| Stage 7<br>21 days | 1 mM N-Acetylcysteine, 10 nM Tri-iodothyronine (T3) and 0.5 μM Aurora kinase inhibitor ZM447439 | CMRL-modified: CMRL 1066 (15-110-CVR, Corning), 2% BSA fV, 2 mM Glutamax, 1:200 ITS-X, Heparin 10 μg/ml, 10 μM Zinc Sulfate, 5 mM Sodium Pyruvate (Lonza), 1:2000 chemically defined lipid concentrate (11905-031, Invitrogen), 1:2000 medium trace elements A (25-021-CI,Cellgro), 1:2000 medium trace elements B (99-175-CI, Cellgro) |

**Supplementary Table 4:** Chemicals and Reagents used in the study

| Chemical/Reagent | Company name | Catalogue# |
|---|---|---|
| Activin A | QKine Ltd, Cambridge, UK | Cat# Qk001 |
| CHIR | Tocris | Cat# 4423 |
| Ascorbic acid | Sigma-Aldrich | Cat# A4544 |
| FGF7 | Genscript | Cat# Z03047 |
| SANT1 | Sigma-Aldrich | Cat# 4572 |
| LDN | Selleckchem | Cat# S2618 |
| Retinoic acid | Sigma-Aldrich | Cat# R2625 |
| TPB | Santa Cruz | Cat# sc-204424 |
| EGF | Peprotech | Cat# AF-100-15 |
| Nicotinamide | Sigma-Aldrich | Cat# N0636 |
| ALK5inhII | Selleckchem | Cat# S7233 |
| GC1 | Tocris | Cat# 4554 |
| GSiXX | Millipore | Cat# 56578 |
| N-Acetylcysteine | Sigma-Aldrich | Cat# A9165 |
| Tri-iodothyronine (T3) | Sigma-Aldrich | Cat# T6397 |
| Aurora kinase inhibitor ZM447439 | Selleckchem | Cat# S1103 |
| Glutamax | Life Technologies | Cat# 35050038 |
| Heparin | Sigma-Aldrich | Cat# H3149 |
| Zinc Sulfate | Sigma-Aldrich | Cat# Z0251 |
| Chemically defined lipid concentrate | Invitrogen | Cat# 11905-031 |
| Medium trace elements A | Cellgro | Cat# 25-021-CI |
| Medium trace elements B | Cellgro | Cat# 99-175-CI |
| TYK2 inhibitor (BMS-986165) | MCE-MedChemExpress | Cat# HY-117287 |
| Human Interferon α 2B | Sigma-Aldrich | Cat# H6166 |
| Recombinant human IFN$\gamma$ | R&D | Cat# 285-IF |

**Supplementary Table 5:** Antibodies used for western blot

| Antibody | Company | Catalog# and clone | Dilution |
|---|---|---|---|
| pSTAT1 (Tyr701) Rabbit mAb | Cell Signaling Technology | Cat# 7649; (D4A7) | 1:1000 |
| STAT1 Mouse mAb | Santa-Cruz | Cat# sc-464; (C-136) | 1:500 |
| pSTAT2 (Tyr690) Rabbit mAb | Cell Signaling Technology | Cat# 88410; (D3P2P) | 1:1000 |
| STAT2 Rabbit mAb | Cell Signaling Technology | Cat# 72604; (D97JL) | 1:1000 |
| pSTAT3 (Tyr705) Rabbit mAb | Cell Signaling Technology | Cat# 9145; (D3A7) | 1:1000 |
| STAT3 Mouse mAb | Cell Signaling Technology | Cat# 9193; (124H6) | 1:1000 |
| TYK2 Mouse mAb | Abcam | Cat# ab57678 | 1:500 |
| K-RAS Mouse mAb | Santa-Cruz | Cat# sc-517599; (3B10-2F2) | 1:250 |
| Tubulin Mouse mAb | Sigma-Aldrich | Cat# T5168; (B-5-1-2) | 1:2000 |
| β-actin-HRP Mouse mAb | Santa Cruz | Cat# sc-47778; (C4) | 1:1000 |

**Supplementary Table 6:** Antibodies used for flow cytometry and immunofluorescence

| Antibody | Company | Catalog# | Dilution |
|---|---|---|---|
| Mouse Anti-CD184 (CXCR4) Monoclonal Antibody | BD Biosciences | Cat# 555974; RRID: AB_396267 | 1:10 |
| Mouse IgG2a, kappa Isotype Control, Phycoerythrin Conjugated | BD Biosciences | Cat# 5555749; RRID: AB_396091 | 1:10 |
| Rabbit anti-OCT4 Rabbit | Santa Cruz Biotechnology | Cat# sc-9081; RRID: AB_2167703 | 1:250 |
| Rabbit anti-SOX2 mAb | Cell Signaling Technology | Cat# 3579; RRID: AB_2195767 | 1:500 |
| Sheep anti Human Neurogenin-3 antibody | R&D systems | Cat# AF3444; RRID: AB_2149527 | 1:400 |
| PE-mouse anti PDX1 | BD Biosciences | Cat# 562161; RRID: AB_10893589 | 1:80 |
| Alexa Fluor 647 Mouse anti NKX6-1 | BD Biosciences | Cat# 563338; RRID: AB_2738144 | 1:80 |
| Alexa Fluor 647 Mouse IgG1 k isotype control | BD Biosciences | Cat# 557714; RRID: AB_396823 | 1:80 |
| Insulin (C27C9) Rabbit Antibody (Alexa Fluor 647 Conjugate) | Cell Signaling Technology | Cat# 9008; RRID: AB_2687822 | 1:80 |
| Rabbit IgG Isotype Control (Alexa Fluor 647 Conjugate) | Cell Signaling Technology | Cat# 3452S; RRID: AB_10695811 | 1:80 |
| Mouse anti-GCG antibody | Sigma-Aldrich | Cat# G2654; RRID: AB_259852 | FC-1:160 ICC- 1:500 |
| Guinea pig anti-INS | Dako | Cat# A0564; RRID: AB_10013624 | 1:1000 |
| Rabbit anti-Ki67 | Leica Microsystems | Cat# NCL-Ki67p; RRID: AB_442102 | 1:500 |
| Mouse anti-MHC Class I monoclonal antibody (W6/32) | Enzo Life Sciences | Cat# ALX-805-711-C100; RRID: AB_11179235 | FC- 1:80 ICC- 1:200 |
| Rabbit anti-SLC18A1 | Sigma-Aldrich | Cat# HPA063797; RRID: AB_2685125 | 1:250 |
| Rabbit anti-Somatostatin | Dako | Cat# A0566; RRID: AB_2688022 | 1:500 |

| | | | |
|---|---|---|---|
| Brilliant violet 605 anti-human HLA-A, B, C (W6/32) | BioLegend | Cat# 311432; RRID: AB_2566151 | 1:100 |
| PE anti-human CD274 (B7-H1, PD-L1) (29E.2A3) | BioLegend | Cat# 329706; RRID: AB_940368 | 1:100 |
| Alexa FluoR 488 Donkey anti-Mouse IgG secondary antibody | ThermoFisher Scientific | Cat# A-21202; RRID: AB_141607 | 1:500 |
| Alexa FluoR 594 Goat anti-Guinea Pig IgG secondary antibody | ThermoFisher Scientific | Cat# A-11076; RRID: AB_2534120 | 1:500 |
| Alexa FluoR 594 Donkey anti-Sheep IgG secondary antibody | ThermoFisher Scientific | Cat# A- 11016; RRID: AB_2534083 | 1:500 |
| Alexa FluoR 488 Donkey anti-Rabbit IgG secondary antibody | ThermoFisher Scientific | Cat# A-21206; RRID: AB_2535792 | 1:500 |
| Alexa FluoR 594 Donkey anti-Rabbit IgG secondary antibody | ThermoFisher Scientific | Cat# A-21207; RRID: AB_141637 | 1:500 |
| Alexa FluoR 488 Donkey anti-Mouse IgG secondary antibody | ThermoFisher Scientific | Cat# A-21203; RRID: AB_141633 | 1:500 |
