# Supplementary material for: The type 1 diabetes gene TYK2 regulates β-cell development and its responses to interferon-α
Source: Nat Commun. 2022 Oct 26;13:6363. doi: 10.1038/s41467-022-34069-z (PMC9606380; doi:10.1038/s41467-022-34069-z)
Supplement: Supplementary file 3 — Reporting Summary [file 41467_2022_34069_MOESM3_ESM.pdf]

nature portfolio

Corresponding author(s): Timo Otonkoski, Vikash Chandra

Last updated by author(s): Oct 11, 2022

Reporting Summary

Nature Portfolio wishes to improve the reproducibility of the work that we publish. This form provides structure for consistency and transparency in reporting. For further information on Nature Portfolio policies, see our Editorial Policies and the Editorial Policy Checklist.

Statistics

For all statistical analyses, confirm that the following items are present in the figure legend, table legend, main text, or Methods section.

n/a	Confirmed	
☐	☒	The exact sample size (*n*) for each experimental group/condition, given as a discrete number and unit of measurement
☐	☒	A statement on whether measurements were taken from distinct samples or whether the same sample was measured repeatedly
☐	☒	The statistical test(s) used AND whether they are one- or two-sided<br>*Only common tests should be described solely by name; describe more complex techniques in the Methods section.*
☒	☐	A description of all covariates tested
☐	☒	A description of any assumptions or corrections, such as tests of normality and adjustment for multiple comparisons
☐	☒	A full description of the statistical parameters including central tendency (e.g. means) or other basic estimates (e.g. regression coefficient) AND variation (e.g. standard deviation) or associated estimates of uncertainty (e.g. confidence intervals)
☐	☒	For null hypothesis testing, the test statistic (e.g. *F*, *t*, *r*) with confidence intervals, effect sizes, degrees of freedom and *P* value noted<br>*Give P values as exact values whenever suitable.*
☒	☐	For Bayesian analysis, information on the choice of priors and Markov chain Monte Carlo settings
☒	☐	For hierarchical and complex designs, identification of the appropriate level for tests and full reporting of outcomes
☐	☒	Estimates of effect sizes (e.g. Cohen's *d*, Pearson's *r*), indicating how they were calculated

Our web collection on statistics for biologists contains articles on many of the points above.

Software and code

Policy information about availability of computer code

Data collection	BD Cellquest Pro v4.0.2, Axio Scan Blue Zeiss software Zen2 Blue Edition v2 and Image Lab v6.0.0.
Data analysis	GraphPad Prism 8.4.2, CellProfiler 4.0, ImageJ 1.53f, FlowJo v10, 10x Genomics Cell Ranger v3.1, DropletUtils v1.6.1, Seurat v3.2.3, Harmony v1.0, Monocle2 v2.14.0, Soupx v1.4.8, STAR alinger v2.5.4b, DESeq2 v1.2.17, Fdrtool v1.2.17, ClusterProfiler v3.18.1, Cutadapt v2.6, Samtools v1.7, Rsubread v2.4.3, ComplexHeatmap v2.6.2, ReactomePA v1.34.0, limma v3.46.0 and ggplot v3.3.5, COMBAT function sva R package v3.32.1) and EdgeR (v3.26.8).

For manuscripts utilizing custom algorithms or software that are central to the research but not yet described in published literature, software must be made available to editors and reviewers. We strongly encourage code deposition in a community repository (e.g. GitHub). See the Nature Portfolio guidelines for submitting code & software for further information.

Data

Policy information about availability of data

All manuscripts must include a data availability statement. This statement should provide the following information, where applicable:

- Accession codes, unique identifiers, or web links for publicly available datasets
- A description of any restrictions on data availability
- For clinical datasets or third party data, please ensure that the statement adheres to our policy

Ultra-deep bulk RNAseq data for pancreatic differentiation stages 1, 4 and 7 of HEL46.11 and for stages 0, 4, 5 and 6 of WT and TYK2 KO genotypes are deposited in the Gene Expression Omnibus database with accession code GSE190727 and GSE190725 which are publicly accessible.
Single-cell RNA-seq data for the WT and TYK2 KO genotypes at pancreatic differentiation stages 5, 6 and IFN alpha treated stage 6 are deposited in the Gene Expression Omnibus database with accession code GSE190726 which is publicly accessible.

Original western blot images are deposited in the Source Data file and at Mendeley: https://data.mendeley.com/datasets/8n9nytgy57/1
All other data are available from the Source Data files and the authors.

Field-specific reporting

Please select the one below that is the best fit for your research. If you are not sure, read the appropriate sections before making your selection.

- [x] Life sciences
- [ ] Behavioural & social sciences
- [ ] Ecological, evolutionary & environmental sciences

For a reference copy of the document with all sections, see nature.com/documents/nr-reporting-summary-flat.pdf

Life sciences study design

All studies must disclose on these points even when the disclosure is negative.

Sample size

Stem cell derived tissue was tested with adequate repeats to confirm consistent data between parallel experimental setups. The range of biological repeats was therefore between n=3 to n=6 depending on availability of material and inherent variability within each assay.

Data exclusions

scRNAseq datasets excluded cell data with low reads or markers of high mitochondrial stress.

Replication

SC-islet differentiations were carried out by at least 2-3 independent researchers utilising the same protocol, and all produced SC-islet batches with similar levels of functionality and measurements between the multiple assays within the study. The number of SC-islet experiments used for each assay is detailed in the figure legends and up to 6 independent experiments including two independent verified TYK2 knockout clones C10 and C12 were used. No SC-islet experiments were excluded from analysis, unless a technical issue with data collection occurred. The differentiation protocol to SC-islets has quality control checkpoints ensuring the robustness of the protocol. The results were reproducible in all the independent experiments performed.

Randomization

No interventions requiring randomization were used for the study. When choosing SC-islets for various analyses, the samples were collected randomly from the whole batch.

Blinding

IHC immunostaining and quantification was blinded through processing batches simultaneously in the CellProfiler pipeline or double-blinded quantification using ImageJ software. Data analysis was performed in a simultaneous and unbiased manner for all collected samples, where no blinding was necessary.

Reporting for specific materials, systems and methods

We require information from authors about some types of materials, experimental systems and methods used in many studies. Here, indicate whether each material, system or method listed is relevant to your study. If you are not sure if a list item applies to your research, read the appropriate section before selecting a response.

Materials & experimental systems

n/a	Involved in the study	
[ ]	[x]	Antibodies
[ ]	[x]	Eukaryotic cell lines
[x]	[ ]	Palaeontology and archaeology
[ ]	[x]	Animals and other organisms
[ ]	[x]	Human research participants
[x]	[ ]	Clinical data
[x]	[ ]	Dual use research of concern

Methods

n/a	Involved in the study	
[x]	[ ]	ChIP-seq
[ ]	[x]	Flow cytometry
[x]	[ ]	MRI-based neuroimaging

Antibodies

Antibodies used

Antibodies used in WB:
- pSTAT1 (Tyr701) Rabbit mAb; Cell Signaling Technology Cat# 7649; (D4A7); Dilution 1:1000
- STAT1 Mouse mAb; Santa-Cruz Cat# sc-464; (C-136); Dilution 1:500
- pSTAT2 (Tyr690) Rabbit mAb; Cell Signaling Technology Cat# 88410; (D3P2P); Dilution 1:1000
- STAT2 Rabbit mAb; Cell Signaling Technology Cat# 72604; (D97JL); Dilution 1:1000
- pSTAT3 (Tyr705) Rabbit mAb; Cell Signaling Technology Cat# 9145; (D3A7); Dilution 1:1000
- STAT3 Mouse mAb; Cell Signaling Technology Cat# 9193; (124H6); Dilution 1:1000
- TYK2 Mouse mAb; Abcam Cat# ab57678; Dilution 1:500
- Tubulin Mouse mAb; Sigma-Aldrich Cat# T5168; (B-5-1-2); Dilution 1:2000
- β-actin-HRP Mouse mAb; Santa Cruz Cat# sc-47778; (C4) Dilution 1:1000
- TYK2 Mouse mAb; Abcam Cat# ab57678; Dilution 1:500
- K-RAS Mouse mAb; Santa-Cruz Cat# sc-517599; (3B10-2F2); Dilution 1:250

Antibodies used for flow cytometry and Immunofluorescence:
- Mouse Anti-CD184 (CXCR4) Monoclonal Antibody BD Biosciences Cat# 555974; RRID: AB_396267; Dilution 1:10
- Mouse IgG2a, kappa Isotype Control, Phycoerythrin Conjugated BD Biosciences Cat# 5555749; Dilution 1:10
- OCT4 Rabbit Santa Cruz Biotechnology Cat# sc-9081; RRID: AB_2167703; Dilution 1:250
- SOX2 Rabbit mAb Cell Signaling Technology Cat# D6D9; Dilution 1:500
- Human Neurogenin-3 antibody R & D systems Cat# AF3444; Dilution 1:400
- PE-mouse anti PDX1 BD Biosciences Cat# 562161; Dilution 1:80
- Alexa Fluor 647-mouse anti NKX6-1 BD Biosciences Cat# 563338; Dilution 1:80
- Alexa Fluor -647 Mouse IgG1 k isotype control BD Biosciences Cat# 557714; Dilution 1:80
- Insulin (C27C9) Rabbit Antibody (Alexa Fluor 647 Conjugate) Cell Signaling Technology Cat# 9008; RRID: AB_2687822; Dilution 1:80
- Rabbit IgG Isotype Control (Alexa Fluor 647 Conjugate) Cell Signaling Technology Cat# 3452S; RRID:AB_10695811; Dilution 1:80
- Mouse anti-GCG unconjugated antibody Sigma-Aldrich Cat# G2654; RRID: AB_259852; Dilution FC-1:160; ICC- 1:500
- Guinea pig anti-INS Dako Cat# A0564; RRID: AB_10013624; Dilution 1:1000
- Rabbit anti-KI67 Leica Microsystems Cat# NCL-Ki67p; RRID: AB_442102; Dilution 1:500
- Mouse anti-MHC Class I monoclonal antibody (W6/32) Enzo Life Sciences Cat# ALX-805-711-C100; RRID: AB_11179235; Dilution FC-1:80; ICC- 1:200
- Brilliant violet 605 anti-human HLA A, B, C (W6/32) BioLegend Cat# 311432; RRID: AB_2566151; Dilution 1:100
- PE anti-human CD274 (B7-H1, PDL1) (29E.2A3) BioLegend Cat# 329706; RRID: AB_940368; Dilution 1:100
- Rabbit anti-SLC18A1 Sigma-Aldrich Cat# HPA063797; RRID: AB_2685125; Dilution 1:250
- Rabbit anti-Somatostatin Dako Cat# A0566; RRID: AB_2688022; Dilution 1:500

Secondary antibodies; Dilution 1:500
- Alexa FluoR 488 Donkey anti-Mouse IgG secondary antibody ThermoFisher Scientific Cat# A-21202; RRID: AB_141607
- Alexa FluoR 594 Goat anti-Guinea Pig IgG secondary antibody ThermoFisher Scientific Cat# A-11076; RRID: AB_2534120
- Alexa FluoR 594 Donkey anti-Sheep IgG secondary antibody ThermoFisher Scientific Cat# A- 11016; RRID: AB_2534083
- Alexa FluoR 488 Donkey anti-Rabbit IgG secondary antibody ThermoFisher Scientific Cat# A-21206; RRID: AB_2535792
- Alexa FluoR 594 Donkey anti-Rabbit IgG secondary antibody ThermoFisher Scientific Cat# A-21207; RRID: AB_141637
- Alexa FluoR 488 Donkey anti-Mouse IgG secondary antibody ThermoFisher Scientific Cat# A-21203; RRID: AB_141633

Validation

All antibodies used were validated either using primary islet tissue or in-house tissue samples or referred the references on the corresponding supplier product Data sheet/company website. E.g. The insulin-AF647 antibody has flow cytometry validation data ("tested in-house for direct immunofluorescent analysis in rat cells and flow cytometry in mouse cells") and relevant citations on the Cell Signaling Technology website. The insulin antibody (DAKO) has numerous citations of use for specific immunohistochemical staining. The glucagon antibody has IF validation images on the Sigma-Aldrich website as well as supplied relevant citations. The Ki-67 antibody has relevant immunohistochemical staining data on the product website (Leica). The TYK2 antibody from Abcam for western blotting was also validated with the TYK2 knockout hiPSCs clones in our study as well as the antibodies for the downstream pSTATs.

Antibodies validation:
• pSTAT1 (Tyr701) Rabbit mAb
Specificity / Sensitivity: Phospho-Stat1 (Tyr701) (D4A7) Rabbit mAb recognizes endogenous levels of Stat1 protein only when phosphorylated at Tyr701.
Species Reactivity: Human, Mouse, Rat.
MW (kDa): 84,91.
Applications: WB-Western Blot.
Validation:
https://www.cellsignal.com/products/primary-antibodies/phospho-stat1-tyr701-d4a7-rabbit-mab/7649
Western blot analysis of extracts from HeLa, A20, and PC-12 cells, untreated or treated with Human Interferon-α1 (hIFN-α1) #8927 (10 ng/ml, 30 min), using Phospho-Stat1 (Tyr701) (D4A7) Rabbit mAb.

• TYK2 Mouse mAb
Specificity / Sensitivity: Recombinant fragment: PVCHLRLLAQ AEGEPCYIRD SGVAPTDPGP ESAAGPPTHEVLVTGTGGIQ WWPVEEEVNK EEGSSGSSGR NPQASLFGKK AKAHKAVGQP
ADRPREPLWA, corresponding to amino acids 276-375 of Human TYK2.
Species Reactivity: Human.
MW (kDa): 134.
Applications: WB.
Validation:
https://www.abcam.com/tyk2-antibody-ab57678.html
ab57678 was shown to specifically react with TYK2 when TYK2 knockout samples were used. Wild-type and ProteinX knockout samples were subjected to SDS-PAGE.
The antibody was also validated in this study using a human stem cell knockout model (Fig. 2a)

• K-RAS Mouse mAb
Specificity / Sensitivity: Raised against full length recombinant K-Ras of human origin.
Species Reactivity: Human.
MW (kDa): 21.
Applications: WB.
Validation:
https://www.scbt.com/p/k-ras-antibody-3b10-2f2
K-Ras Antibody (3B10-2F2): sc-517599. Western blot analysis of K-Ras expression in non-transfected: sc-110760and human K-Ras

transfected: sc-111225 whole cell lysates.

• STAT1 Mouse mAb
Specificity / Sensitivity: Stat1 (C-136) is a mouse monoclonal antibody raised against amino acids 613-739 of Stat1 of human origin.
Species Reactivity: Mouse, rat and human.
MW (kDa): 91/84.
Applications: WB.
Validation:
https://www.scbt.com/p/stat1-antibody-c-136?requestFrom=search
Stat1 Antibody (C-136): sc-464. Western blot analysis of Stat1 expression in non-transfected 293T: sc-117752, human Stat1 transfected 293T: sc-177983 and HeLa whole cell lysates.

• pSTAT2 (Tyr690) Rabbit mAb
Specificity / Sensitivity: Phospho-Stat2 (D3P2P) Rabbit mAb recognizes endogenous levels of Stat2 protein only when phosphorylated at Tyr690.
Species Reactivity: Mouse, rat and human.
MW (kDa): 97, 113.
Applications: WB.
Validation:
https://www.cellsignal.com/products/primary-antibodies/phospho-stat2-tyr690-d3p2p-rabbit-mab/88410
Western blot analysis of extracts from serum-starved U266 and A-431 cells, untreated or treated with Human Interferon-α1 (hIFN-α1) #8927 (10 ng/ml; +) using Phospho-Stat2 (Tyr690) (D3P2P) Rabbit mAb and total Stat2 (D9J7L) Rabbit mAb #72604.

• STAT2 Rabbit mAb
Specificity / Sensitivity: Monoclonal antibody is produced by immunizing animals with a synthetic peptide corresponding to residues surrounding Leu706 of human Stat2 protein.
Species Reactivity: Human and mouse.
MW (kDa): 97, 113.
Applications: WB.
Validation:
https://www.cellsignal.com/products/primary-antibodies/stat2-d9j7l-rabbit-mab/72604
Western blot analysis of extracts from various cell lines using Stat2 (D9J7L) Rabbit mAb. KARPAS cell line source: Dr Abraham Karpas at the University of Cambridge.

• pSTAT3 (Tyr705) Rabbit mAb
Specificity / Sensitivity: Phospho-Stat3 (Tyr705) (D3A7) XP® Rabbit mAb detects endogenous levels of Stat3 only when phosphorylated at tyrosine 705. This antibody does not cross-react with phospho-EGFR or the corresponding phospho-tyrosines of other Stat proteins.
Species Reactivity: Human, mouse, rat and monkey.
MW (kDa): 79, 86.
Applications: WB.
Validation:
https://www.cellsignal.com/products/primary-antibodies/phospho-stat3-tyr705-d3a7-xp-rabbit-mab/9145
Western blot analysis of extracts from IFN-alpha treated Jurkat cells and HeLa cells, as well as EGF treated A431 cells, using Phospho-Stat3 (Tyr705) (D3A7) XP® Rabbit mAb. Note that the basal phospho-Stat3 in A431 is detected by the antibody.

• STAT3 Mouse mAb
Specificity / Sensitivity: Monoclonal antibody is produced by immunizing animals with a synthetic peptide centered around amino acid Gln692 of human Stat3.
Species Reactivity: Human, mouse, rat and monkey.
MW (kDa): 79, 86.
Applications: WB.
Validation:
https://www.cellsignal.com/products/primary-antibodies/stat3-124h6-mouse-mab/9139
Western blot analysis of extracts from HeLa, NIH/3T3, PC12 and COS cells, using Stat3 (124H6) Mouse mAb.

• Tubulin Mouse mAb
Specificity / Sensitivity: Monoclonal Anti-α-Tubulin (mouse IgG1 isotype) is derived from the B-5-1-2 hybridoma produced by the fusion of mouse myeloma cells and splenocytes from an immunized mouse. Sarkosyl-resistant filaments from Strongylcentrotus purpuratus (sea urchin) sperm axonemes1 were used as the immunogen.
Species Reactivity: Chicken, sea urchin, rat, Chlamydomonas, bovine, human, African green monkey, mouse.
MW (kDa): 50.
Applications: WB.
Validation:
https://www.sigmaaldrich.com/FI/en/product/sigma/t5168
Immunoblotting. Cell line lysates were separated on SDS-PAGE and probed with 1:4,000 Monoclonal Anti-a-
Tubulin Clone: B-5-1-2 (Cat. No.T5168). The antibody was developed using Goat Anti-Mouse
IgG-Peroxidase (Cat. No. A2304) and a chemiluminescent substrate. Tested on Hela, JURKAT, COS7, NIH-3T3, PC-12, RAT2, CHO, MDBK and MDCK cell-lines.

• β-actin-HRP Mouse mAb
Specificity / Sensitivity: β-Actin (C4) is a mouse monoclonal antibody raised against gizzard Actin of chicken origin.
Species Reactivity: Mouse, rat, human, avian, bovine, canine, porcine, rabbit, Dictyostelium discoideum and Physarum polycephalum.
MW (kDa): 43.
Applications: WB.
Validation:
https://www.scbt.com/p/beta-actin-antibody-c4
β-Actin (C4): sc-47778. Western blot analysis of β-Actin expression in HeLa, Jurkat and NIH/3T3 whole cell lysates and human spleen tissue extract.

• Mouse Anti-CD184 (CXCR4) Monoclonal Antibody
Specificity / Sensitivity: SIVmac variant CP-MAC-infected Sup-T1 cells
Species Reactivity: Human (QC Testing), Rhesus, Cynomolgus, Baboon (Tested in Development).
Applications: Flow cytometry.
Validation:
https://www.bdbiosciences.com/en-us/products/reagents/flow-cytometry-reagents/research-reagents/single-color-antibodies-ruo/pe-mouse-anti-human-cd184.555974
Flow cytometric analysis of CD184 expression on human peripheral blood lymphocytes. Whole blood was stained with either PE Mouse IgG2a, κ Isotype Control (Cat. No. 555574; dashed line histogram) or PE Mouse Anti-Human CD184 (Cat. No. 555974/561733/557145; solid line histogram). Erythrocytes were lysed with BD Pharm Lyse™ Lysing Buffer (Cat. No. 555899). Fluorescent histograms were derived from gated events with the side and forward light-scattering characteristics of viable lymphocytes.

• Mouse IgG2a, kappa Isotype Control, Phycoerythrin Conjugated
Specificity / Sensitivity: The MOPC-21 immunoglobulin is a mouse myeloma protein. The MOPC-21 immunoglobulin was selected as an isotype control following screening for low background on a variety of mouse and human tissues.
Species Reactivity: Human and mouse.
Applications: Flow cytometry.
Validation:
https://www.bdbiosciences.com/en-fi/products/reagents/flow-cytometry-reagents/research-reagents/flow-cytometry-controls-and-lysates/pe-mouse-igg1-isotype-control.555749
Profile of peripheral blood lymphocytes analyzed on a FACScan (BDIS, San Jose, CA)

• Rabbit anti-OCT4 Rabbit
Specificity / Sensitivity: Oct-3/4 (H-134) is a rabbit polyclonal antibody raised against amino acids 1-134 mapping at the N-terminus of Oct-3/4 of human origin.
Species Reactivity: Mouse, rat and human.
Applications: IF.
Validation:
https://pubmed.ncbi.nlm.nih.gov/26293300/
Immunofluorescence analysis of human or mouse blastocysts for Oct4/OCT4.

• Rabbit anti-SOX2 mAb
Specificity / Sensitivity: Monoclonal antibody is produced by immunizing animals with a synthetic peptide corresponding to amino acids surrounding Gly179 of human Sox2.
Species Reactivity: Human.
Applications: IF.
Validation:
https://www.cellsignal.com/products/primary-antibodies/sox2-d6d9-xp-rabbit-mab/3579
Confocal immunofluorescent analysis of NTERA2 (left) and HeLa (right) cells using Sox2 (D6D9) XP® Rabbit mAb (green). Actin filaments have been labeled with DY-554 phalloidin (red).

• Sheep anti Human Neurogenin-3 antibody
Specificity / Sensitivity: Detects human Neurogenin-3 in direct ELISAs and Western blots. In direct ELISAs, approximately 10% cross-reactivity with recombinant mouse Neurogenin-3 is observed, and less than 1% cross-reactivity with recombinant human Neurogenin-1 is observed. immunogen is E. coli-derived recombinant human Neurogenin-3 (Met1-Leu214)
Species Reactivity: Human.
Applications: IF.
Validation:
https://www.rndsystems.com/products/human-neurogenin-3-antibody_af3444?gclid=CjwKCAjwsfuYBhAZEiwA5a6CDKTPpWZ13BmI5tAGbJtfKHbOC8gVbSUrGcMKNMk9iZGbBPeLPpV_GxoCbiMQAvD_BwE&gclsrc=aw.ds#product-datasheets
Neurogenin-3 was detected in immersion fixed paraffin-embedded sections of human placenta.

• PE-mouse anti PDX1
Specificity / Sensitivity: The 658A5 monoclonal antibody binds PDX-1 or Pancreas/Duodenum Homeobox Protein-1 encoded by the PDX1 gene.
Species Reactivity: Mouse and human.
Applications: Flow cytometry.
Validation:

https://www.bdbiosciences.com/en-us/products/reagents/flow-cytometry-reagents/research-reagents/single-color-antibodies-ruo/pe-mouse-anti-pdx-1.562161
Flow cytometric analysis of PDX-1 in mouse pancreatic tumor (insulinoma) cells Beta-TC-6.

• Alexa Fluor 647 Mouse anti NKX6-1
Specificity / Sensitivity: The R11-560 monoclonal antibody specifically binds to human and mouse homeobox protein Nkx6.1.
Species Reactivity: Mouse and human.
Applications: Flow cytometry.
Validation:
https://www.bdbiosciences.com/en-fr/products/reagents/flow-cytometry-reagents/research-reagents/single-color-antibodies-ruo/alexa-fluor-647-mouse-anti-nkx6-1.563338
Flow cytometric analysis of Nkx6.1 expression in mouse pancreatic tumor (insulinoma) cells Beta-TC-6.

• Alexa Fluor 647 Mouse IgG1 k isotype control
Specificity / Sensitivity: The MOPC-21 immunoglobulin is a mouse myeloma protein. The MOPC-21 immunoglobulin was selected as an isotype control following screening for low background on a variety of mouse and human tissues.
Species Reactivity: Mouse and human.
Applications: Flow cytometry.
Validation:
https://www.bdbiosciences.com/en-eu/products/reagents/flow-cytometry-reagents/research-reagents/flow-cytometry-controls-and-lysates/alexa-fluor-647-mouse-igg1-isotype-control.557783
Flow cytometric analysis on U-937 cells.

• Insulin (C27C9) Rabbit Antibody (Alexa Fluor 647 Conjugate)
Specificity / Sensitivity: Insulin (C27C9) Rabbit mAb detects endogenous levels of total insulin protein.
Species Reactivity: Human, mouse and rat.
Applications: Flow Cytometry.
Validation:
https://www.cellsignal.com/products/primary-antibodies/insulin-c27c9-rabbit-mab/3014
Flow cytometric analysis of C2C12 cells and β-TC-6 cells using Insulin (C27C9) Rabbit mAb or a concentration-matched Rabbit (DA1E) mAb IgG XP® Isotype Control #3900.

• Rabbit IgG Isotype Control (Alexa Fluor 647 Conjugate)
Specificity / Sensitivity: The purified antibody is not directed against any known antigen. It functions as an isotype control for rabbit antibodies.
Species Reactivity: Human and mouse.
Applications: Flow cytometry.
Validation:
https://www.cellsignal.com/products/antibody-conjugates/rabbit-igg-isotype-control-alexa-fluor-647-conjugate/3452
Flow cytometric analysis of Jurkat cells, untreated or etoposide-treated, using Phospho-Histone H2A.X (Ser139) (20E3) Rabbit mAb (Alexa Fluor® 647 Conjugate) #9720 compared to Rabbit IgG Isotype Control (Alexa Fluor® 647 Conjugate).

• Mouse anti-GCG antibody
Specificity / Sensitivity: Monoclonal Anti-Glucagon antibody produced in mouse against polymerized porcine glucagon, clone K79bB10, ascites fluid.
Species Reactivity: Mouse and human,
Applications: Immunohistochemistry, immunofluorescence and flow cytometry.
Validation:
https://www.sigmaaldrich.com/FI/en/product/sigma/g2654
Immunofluorescence, mouse pancreatic islet alpha cells were stained using Monoclonal Anti-Glucagon, from Yuying Jiang, Columbus Children's Research Institute, Columbus, OH.

• Guinea pig anti-INS
Specificity / Sensitivity: Polyclonal guinea pig Anti-Porcine pancreatic insulin.
Species Reactivity: The antibody cross-reacts with insulin from several mammalian species. Specificity as determined by radioimmunoassay was 100% for human insulin, 100% for porcine insulin and less than 0.05% for glucagon and human growth hormone. This product has been optimized for use on human tissues.
Applications:
Validation:
https://www.agilent.com/en/product/immunohistochemistry/antibodies-controls/primary-antibodies/insulin-%28autostainer-link-48%29-76277#specifications
Pancreas immunostaining. The Beta cells in the islets of Langerhans show a moderate to strong cytoplasmic staining reaction. Clinical application for IR002/IS002 For identification of insulin-producing cells in normal and neoplastic tissue.

• Rabbit anti-Ki67
Specificity / Sensitivity: Human Ki67 nuclear antigen expressed in all proliferating cells during late G1, S, G2 and M phases of the cell cycle. Immunogen: Prokaryotic recombinant fusion protein corresponding to 1086 bp Ki67 motif-containing cDNA fragment.
Species Reactivity: Common marmoset, human, mouse, rat and zebrafish.
Applications: Immunohistochemistry, immunocytochemistry, immunohistochemistry - paraffin section.
Validation:

https://www.yumpu.com/en/document/read/182377/novocastratm-lyophilized-rabbit-polyclonal-antibody-ki67-antigen
Nat Commun. 2021 Jun 17;12(1):3707. doi: 10.1038/s41467-021-23973-5.
Photomicrographs of Ki-67 (a) and BrdU (c) stainings of Braf-Pten-Brn2-WT/het/hom tumors, showing increased proportion of Ki-67 positive cells within tumors.

• Mouse anti-MHC Class I monoclonal antibody (W6/32)
Specificity / Sensitivity: Recognizes virtually all nucleated human cells. Suitable as positive control for HLA tissue typing and crossmatching.
Species Reactivity: Human, Bovine, Cat and Monkey. Does not cross-react with rabbit MHC class I.
Applications: Flow Cytometry, ICC.
Validation:
Immunogenetics.1999 Apr;49(4):312-20. doi: 10.1007/s002510050498.
The W6/32negative cell line OM 531H was transfected with AotusB2m*2, the variant obtained from the W6/32-positive cell line OM VI-3. The transfected cells were tested for binding of the W6/32 monoclonal antibody by flow cytometry. The cells transfected with Aotus-B2m*2 bound W6/32, whereas the cells transfected with vector alone did not.

• Rabbit anti-SLC18A1
Specificity / Sensitivity: SLC18A1 (solute carrier family 18 member A1) is also termed as vesicular monoamine transporter 1 (VMAT1). It is expressed in brain and in neurons of the peripheral nervous system, endocrine tissue and chromaffin cells.
Species Reactivity: Human.
Applications: IHC.
Validation:
https://www.sigmaaldrich.com/FI/en/product/sigma/hpa063797
Immunohistochemistry analysis in human adrenal gland and pancreas tissues using HPA063797 antibody. Corresponding SLC18A1 RNA-seq data are presented for the
same tissues.

• Rabbit anti-Somatostatin
Specificity / Sensitivity:
Species Reactivity: Human, mouse, rat and zebrafish.
Applications: Immunohistochemistry, immunocytochemistry, immunohistochemistry - paraffin section.
Validation:
Nature. 2016 Mar 3;531(7592):105-9. doi: 10.1038/nature16951. Epub 2016 Feb 10.
Immunofluorescence of human ES-cell-derived enteric neuron subtypes.

Am J Surg Pathol. 2015 May;39(5):592-601. doi: 10.1097/PAS.0000000000000383
Immunohistochemically stained slides of each hormone-labeled NET showing: Insulin, GLP1, glucagon, gastrin, somatostatin, and serotonin expression.

• Brilliant violet 605 anti-human HLA-A, B, C (W6/32)
Specificity / Sensitivity: Recognizes virtually all nucleated human cells. Suitable as positive control for HLA tissue typing and crossmatching.
Species Reactivity: Human, Cynomolgus, Rhesus.
Applications: Flow cytometry.
Validation:
https://www.biolegend.com/ja-jp/products/brilliant-violet-605-anti-human-hla-a-b-c-antibody-12842
Human peripheral blood lymphocytes were stained with HLA-A,B,C (clone W6/32) Brilliant Violet 605™ (filled histogram) or mouse IgG2a, κ Brilliant Violet 605™ isotype control (open histogram).

• PE anti-human CD274 (B7-H1, PD-L1) (29E.2A3)
Specificity / Sensitivity: Programmed cell death ligand 1 (PD-L1), B7 homolog 1 (B7-H1).
Species Reactivity: African Green, Baboon, Cynomolgus, Rhesus.
Applications: Flow cytometry.
Validation:
https://www.biolegend.com/nl-be/products/pe-anti-human-cd274-b7-h1-pd-l1-antibody-4375
PHA-stimulated (3 days) human peripheral blood lymphocytes were stained with CD274 (clone 29E.2A3) PE (filled histogram) or mouse IgG2b, κ PE isotype control (open histogram).

J Immunol February 1, 2003, 170 (3) 1257-1266; DOI: https://doi.org/10.4049/jimmunol.170.3.1257
Anti-PD-L1 and anti-PD-L2 mAbs react specifically with PD-L1 and PD-L2, respectively, and block binding of PD-1-Ig. A, 300.19 cells transfected with human PD-L1 or PD-L2 or untransfected were stained with anti-PD-L1 and anti-PD-L2 mAbs. Untransfected 300.19 cells and isotype-matched mAbs were used as negative controls.

Eukaryotic cell lines

Policy information about cell lines

Cell line source(s)	H1 human embryonic stem cells (hESCs) were purchased from WiCell. HEL46.11 iPSC line was generated and characterized in the Biomedicum Stem Cell Center (BSCC) core facility in the University of Helsinki. The knockout clones C10 and C12 were generated in our lab using CRISPR-Cas9-mediated genome editing as described in the methods section.
Authentication	De novo iPSC lines pluripotency and genomic stability were validated by the BSCC core facility with mehods including G-band karyotyping, immunohistochemistry and quantitative PCR for pluripotency markers (OCT4, NANOG, TRA1-60, SOX2, SSEA4), teratoma assays and Promega StemElite STR ID system. The authenticated HEL46.11 were reported in Trokovic et. al 2015.
Mycoplasma contamination	The cell-lines used were routinely tested negative for mycoplasma.
Commonly misidentified lines (See ICLAC register)	Not used

Animals and other organisms

Policy information about studies involving animals; ARRIVE guidelines recommended for reporting animal research

Laboratory animals	Mus Musculus, NOD-Scid-Gamma, Male only, Age 3-8 months at SC-islet implantation. Mice maintained at the Biomedicum Helsinki animal facility on a 12-h light/dark cycle with ad libitum food, 2016 Teklad global 16% protein rodent diets (Envigo). The temperature was kept at 23°C with 24 relative humidity (RH).
Wild animals	No wild animals were used in this study.
Field-collected samples	No field collected samples were used in this study.
Ethics oversight	Animal care and experiments were approved by National Animal Experiment Board in Finland (ESAVI/14852/2018).

Note that full information on the approval of the study protocol must also be provided in the manuscript.

Human research participants

Policy information about studies involving human research participants

Population characteristics	Islets from organ donors were provided by the Nordic Islet Transplantation Program (www.nordicislets.org), Sweden through the Human Tissue Lab (HTL) at LUDC, which is one of the platforms of the EXODIAB network. Excellence of Diabetes Research in Sweden EXODIAB is a joint strategic research initiative in the diabetes area at Lund University (LU) and Uppsala University (UU), and its aim is to facilitate diabetes research globally, for example, by creating resources and tools that can be used by the research community (funding Swedish Research Council: strategic research environment grant (EXODIAB, 2009-1039).<br><br>For the samples collected from foetuses from terminated pregnancies, fetal/embryonic age was determined by measuring crown-rump length and Carnegie stage, where appropriate. Biological sex was inferred from gene expression data.<br><br>For SNP studies, FinnGen (https://www.finngen.fi/en) is a large biobank study aiming to collect and analyse genome and health data from 500,000 Finnish biobank participants. In current study we used R7 dataset of FinnGen project (r7.finngen.fi), which compiled 3095 clinical endpoints obtained from electronic health record data of 309,154 Finnish individuals. The detail biobank based sample collection descriptions and genotyping details can be found in online document https://finngen.gitbook.io/documentation/v/r7/methods/.
Recruitment	Human islets from organ donors were provided by the Nordic Islet Transplantation Program in Lund, Sweden. Human fetal pancreas tissue was dissected from material available following elective termination of pregnancy at the University Hospital in Malmö, Sweden. The study groups for SNP studies are from the general Biobank Finngen.
Ethics oversight	Ethical approval of the collection of human pancreatic islets from organ donors has been obtained from the ethical committee in Lund (Dnr. LU 2011/263). All procedures in human islets were approved by the ethics committees at the Uppsala and Lund Universities and informed consent obtained by appropriate measures from donors or their relatives.<br><br>Ethical permit for samples collected from fetal pancreas has been obtained from the ethical committee in Lund (Dnr2012/593, Dnr 2015/241 and 2018/579). Any information pertaining to the mothers is not accessible and is not documented for research, given the sensitive nature. All procedures regarding human fetal pancreas were approved by the ethics committees at the Lund University and informed consent obtained by appropriate measures from relatives.<br><br>Patients and control subjects in FinnGen provided informed consent for biobank research, based on the Finnish Biobank Act. Alternatively, separate research cohorts, collected prior the Finnish Biobank Act came into effect (in September 2013) and start of FinnGen (August 2017), were collected based on study-specific consents and later transferred to the Finnish biobanks after approval by Fimea, the National Supervisory Authority for Welfare and Health. Recruitment protocols followed the biobank protocols approved by Fimea. The Coordinating Ethics Committee of the Hospital District of Helsinki and Uusimaa (HUS) approved the FinnGen study protocol Nr HUS/990/2017. The FinnGen study is approved by Finnish Institute for Health and Welfare (permit numbers: THL/2031/6.02.00/2017, THL/1101/5.05.00/2017, THL/341/6.02.00/2018, THL/2222/6.02.00/2018, THL/283/6.02.00/2019, THL/1721/5.05.00/2019, THL/1524/5.05.00/2020, and

	THL/2364/14.02/2020), Digital and population data service agency (permit numbers: VRK43431/2017-3, VRK/6909/2018-3, VRK/4415/2019-3), the Social Insurance Institution (permit numbers: KELA 58/522/2017, KELA 131/522/2018, KELA 70/522/2019, KELA 98/522/2019, KELA 138/522/2019, KELA 2/522/2020, KELA 16/522/2020 and Statistics Finland (permit numbers: TK-53-1041-17 and TK-53-90-20).<br><br>The study design and conduct complied with all relevant regulations regarding the use of human study participants and was conducted in accordance with the criteria set by the Declaration of Helsinki.

Note that full information on the approval of the study protocol must also be provided in the manuscript.

Flow Cytometry

Plots

Confirm that:

- [x] The axis labels state the marker and fluorochrome used (e.g. CD4-FITC).
- [x] The axis scales are clearly visible. Include numbers along axes only for bottom left plot of group (a 'group' is an analysis of identical markers).
- [x] All plots are contour plots with outliers or pseudocolor plots.
- [x] A numerical value for number of cells or percentage (with statistics) is provided.

Methodology

Sample preparation	SC-islets were dissociated with TrypLE for 6 minutes at 37°C before single-cell filtering and fixation/permeabilisation in BD Cytofix/CytoPerm solution for 20 minutes at 4°C. Primary antibodies were incubated overnight at 4°C in a 5% FBS CytoPerm buffer and secondary antibodies for a subsequent 45 min at RT. The cells were then analysed using FACSCalibur cytometer (BD Biosciences) and FlowJo software (Tree Star Inc.).
Instrument	BD FACSCalibur (Becton Dickinson)
Software	BD CellQuest Pro v4.0.2 (Acquisition)<br>FlowJo v10 (Analysis)
Cell population abundance	The endocrine precursors cell populations were in this study at stage 5 ranged from 30-60%. The major mature endocrine cell population abundances ranged from 5% to >50% depending on the time of maturation within the experiment.
Gating strategy	For all the flow cytometry samples, cells were gated with FSC and SSC to remove cellular debris. Positive and negative gating was determined through negatively stained cells within the same population and/or non-stained conjugated IgG isotype controls. For the T-cell cytotoxic assay, after excluding CFSEnegCTVneg CD8+ T cells, SC-islet cells were gated on viable singlets and then on CFSE+ and CTV+ fractions to calculate their relative proportion. The gating figures are shown in Fig. 2f, 2i, 6c, 6d, 7b, 7c and Supplementary Fig. 2a, 2b, 5c.

- [x] Tick this box to confirm that a figure exemplifying the gating strategy is provided in the Supplementary Information.
